# Physical, Chemical, and Microbiological Characteristics of Pulque: Management of a Fermented Beverage in Michoacán, Mexico

**DOI:** 10.3390/foods9030361

**Published:** 2020-03-20

**Authors:** Gonzalo D. Álvarez-Ríos, Carmen Julia Figueredo-Urbina, Alejandro Casas

**Affiliations:** 1 Instituto de Investigaciones en Ecosistemas y Sustentabilidad, Universidad Nacional Autónoma de México, Morelia, 58190 Michoacán, Mexico; galvarez7393@gmail.com; 2 Cátedras CONACYT-Laboratorio de Genética, Área Académica de Biología Instituto de Ciencias Básicas e Ingeniería. Universidad Autónoma del Estado de Hidalgo, Mineral de la Reforma, 78557 Hidalgo, Mexico; figueredocj@gmail.com

**Keywords:** agave sap, probiotics, traditional knowledge, biocultural diversity

## Abstract

Pulque is a beverage that has been prepared in Mexico since pre-Hispanic times from the fermented sap of more than 30 species of wild and domesticated agaves. We conducted studies in two communities of the state of Michoacán, in central-western Mexico, where we documented its traditional preparation and analyzed the relationship between preparation conditions and the composition and dynamics of microbiological communities, as well as the physical and chemical characteristics of the beverage. In one of the communities, Santiago Undameo (SU), people boil the sap before inoculating it with pulque inoculum; this action causes this local pulque to be sweeter, less acidic, and poorer in bacteria and yeast diversity than in the other community, Tarimbaro (T), where the agave sap is not boiled and where the pulque has more diversity of microorganisms than in SU. Fermentation management, particularly boiling of the agave sap, influences the dynamics and diversity of microbial communities in the beverage.

## 1. Introduction

Fermentation of fruits, seeds, and other edible substrates is an ancient strategy for obtaining food, involving techniques of biotic resource management and was probably utilized before the advent of agriculture, as suggested by ancient archaeological records in ceramic remains showing the presence of rice ferments in China (about 9000 years BP) and wine in Iran (8000 years BP) and Egypt (5000 years BP) [1,2,3]. Management of fermentation has been crucial for the development of many civilizations, since it allowed for preservation; better digestibility; stable availability; and longer maintenance of the nutritious properties, flavour, and texture, among other features, of food [4,5].

The earliest forms of managing fermentation most likely involved “spontaneous” ferments of fruits and seeds, using strains of microorganisms from the environment [6]. Later on, people generated specialized methods of fermentation in controlled, lowly variable conditions (in terms of competition, nutrient availability, and the bio-physical environment), which made obtaining desirable products possible [7,8]. Management of fermentation has created new niches and contexts in which human selection of microorganisms may operate, consciously or unconsciously [9]. Specialized strains confer characteristics to ferments like nutrients, flavour, texture, colour, smell, and durability, which are directly “selected” by people by seeking the conditions to ensure them [10,11]. In traditional societies, knowledge and practices for preparing fermented products have passed from generation to generation among households and communities, with people sharing some of them but jealously keeping secret others that confer distinctiveness to the producers, thus generating a broad spectrum of product qualities within and among localities [12,13].

Several studies have documented how the diversity and relativeness of microorganism strains are closely related to fermented substrates [5,7,14]. This is the case for *Saccharomyces*, a genus of yeasts participating in fermentation of numerous substrates used by humans throughout history, since they produce non-toxic substances, high levels of alcohol, and compounds important for flavour, like esters and phenols [5,6]. Another group is the lactic acid bacteria (LAB), which occur in numerous substrates and form part of the microbiota of the digestive tract of numerous animal species, including humans [3,7]. The metabolism of these bacteria produce compounds like lactic acid and extracellular polysaccharides (EPS), influencing various qualities of food and beverages [15,16].

Fermented beverages currently have high economic and cultural importance [17,18]. Around the world, there is a wide spectrum of substrates used for this purpose, among them economically important plants whose leaves (e.g., tea), sap (palms; palm wine), fruits (grapes; wine), and grains (barley; beer) are used [6]. In Mexico, numerous cultural groups have used different substrates to prepare fermented beverages, including fruits (e.g., *Spondias* spp. and *Ananas comosus* for preparing “tepache”, *Opuntia* spp. for “colonche”), the sap of plants (*Cocos nucifera* for preparing “tuba”, *Acrocomia aculeata* for “taberna”, *Agave* spp. for “pulque”), grains (*Zea mays* for “tesgüino” or “pozol”), and barks (*Lonchocarpus longistylus* for “balche”) (Table 1) [19,20,21,22,23,24,25].

Pulque is a fermented beverage that has been prepared since pre-Columbian times from sap from 41 taxa of *Agave* in Mexico [28]. The people of Mesoamerica managed fermentation processes; the material evidence of pulque preparation are vessels with remains of this beverage found in residential areas of Teotihuacan, dating back to 1600–1350 BP [29,30]. There is also pictographic evidence, for instance in the Vindobonensis Codex (Mixtec) and in the *“Matrícula de los tributos”* (Nahuatl) [31]. In addition, Sahagún [32] described in *“Historia general de las Cosas de Nueva España”* the methods that the native people of Mexico used to ferment agave sap, the materials and care required, and practices to accelerate the fermentation by adding root plants like the *“Ocpatli”* (*Acacia angustissima*). Such ancient knowledge about the management of fermenting processes has survived in many pulque-producing communities in Mexico.

Agave sap contains sugars (sucrose, fructose and glucose), vitamins, minerals, and amino acids, so it is a source of nutrition [33,34,35,36]. Agave sap has a high diversity of microorganisms, which determine the attributes of pulque [26,34,37,38]. It is rich in different compounds favourable for microorganisms naturally associated with agaves and that are transported to the scraped cavities of the stems through the air or the action of scrapers and other utensils used for collecting agave sap [26,37].

Viscosity, acidity, and alcoholic content are the main attributes determining pulque qualities, and these attributes are conferred by microorganisms participating in the fermentation process. Viscosity is mainly associated with the metabolism of bacteria of the genus *Leuconostoc*, which acidifies the sap using sucrose, producing the CO_2_ and EPS responsible for viscosity [26,39]. 

*Lactobacillus* spp. produce lactic acid, thus increasing acidity and also producing aromatic compounds influencing flavour [3,5,7]. In addition, these bacteria compete with and inhibit growth of pathogenic microbes by producing organic acids and protein toxins called bacteriocins, which inhibit the growth of non-related bacteria [40,41]. Alcoholic production results from yeasts, which in addition produce vitamins and amino acids, as well as volatile compounds that also influence flavour and the aromatic profile of the pulque [6,14,26].

Communities of microorganisms are managed by pulque producers based on their experience and knowledge derived from fermenting agave sap throughout many generations, passing down recipes, ingredients, conditions, and techniques of preparation, which are an important biocultural heritage. Historically, pulque preparation has been artisanal, and in such a context, a great variation of qualities have been recorded, associated with the species and varieties of agave used; environmental conditions of sites where the pulque is prepared; the assemblages of microorganisms available in the producing areas; the cultural contexts guiding preferences for viscosity, acidity, alcoholic content, flavour, and other attributes; and the preparation techniques, including other ingredients added to the sap and the utensils used for collecting, storing, and fermenting the agave sap, among other aspects [26,27].

The state of Michoacán, in central-western Mexico, has been a pulque producing area since Pre-Hispanic times. Agave cultivation in this region is associated with other crops such as maize, squash and beans, in systems that have barely been studied compared with other pulque production systems in Mexico. Álvarez–Ríos et al. [27] documented two pulque producing communities in Michoacán, in which differentiated management techniques are practiced. In this study, we explored the hypothesis that the different management techniques influence the different physical and chemical attributes and microbiological compositions, determining the different features of the produced pulques. This study aimed to document how the techniques of management of agave sap affect the physical and chemical characteristics of the sap and pulque, and the consequences in the structure and dynamics of the consortiums of microorganisms participating in fermentation of this beverage in two communities of Michoacán.

## 2. Materials and Methods 

### 2.1. Study Site

**Figure 1:**
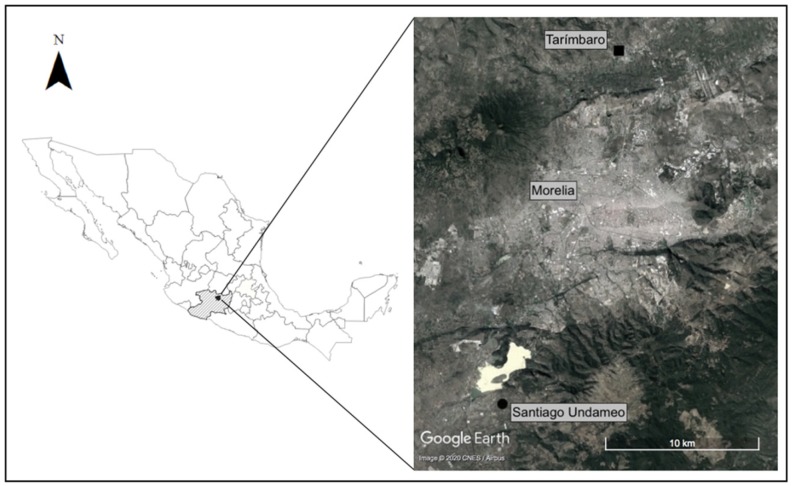
Studied localities in the state of Michoacán. Tarímbaro (T, square) to the north, and Santiago Undameo (SU, circle) to the southwest of the city of Morelia.

Our study was conducted in two localities of the state of Michoacán, central Mexico. One was Tarímbaro (T), north of the city of Morelia, at an elevation of 1860 m, where the annual mean temperature and rainfall are 22 °C and 600–800 mm, respectively. Tarímbaro is part of the suburban area of the city of Morelia, and the main economic activities are agriculture of maize, vegetables, and agave [42]. The other site is Santiago Undameo (SU), southeast of the city of Morelia, at an elevation of 2004 m, with an annual mean temperature and rainfall of 17 °C and 800–1000 mm, respectively. Economic activities in Santiago Undameo are predominantly agriculture of maize, cultivation of agave for preparation of pulque, and cattle raising [42] (Figure 1).

### 2.2. Ethnobiological Fieldwork

A total of 12 in-depth interviews were conducted with households of pulque producers (six in each village) to document management practices of agave sap and preparation of pulque. 

### 2.3. Evaluation of Physical and Chemical Characteristics of Fermentation Phases

We collected samples of the main phases of pulque preparation. In Santiago Undameo, we collected six samples of each of the following fermentation phases of the beverage: l) fresh sap (FS) or *“aguamiel”*, 2) boiled sap (BS), 3) pulque (P), and 4) inoculum for pulque (IP) or “foot of pulque”. Similarly, in Tarímbaro, we collected six samples of the mentioned phases, except the boiled sap since in that community people do not engage in this practice. In SU we collected fresh sap of *A. salmiana* var. *salmiana,* while in T the samples were of *A. mapisaga*. The pulque and inoculum for pulque in both sites were a mixture of sap of three species of *Agave*, as reported by Álvarez-Ríos et al. [27] (*A. salmiana* var. *salmiana, A. mapisaga* and *A. americana*). In total, we collected and analysed 42 samples; 24 from Santiago Undameo and 18 from Tarímbaro.

The following characteristics were evaluated for all collected samples: (1) Concentration of sugars in sap and pulque, measured through a manual refractometer Vee Gee, ABT-32. (2) Acidity, evaluated through a pH meter Denver Instrument, model 215. (3) Lactic acid: to 10 mL of each sample we added 1 mL of 2% phenolphthalein, then titrated with a solution of 0.1 N NaOH. Based on the amount of NaOH used for neutralizing the acid solution, we estimated the total acidity of the sample in grams of lactic acid using the formula *TA=* (*v × N × 0.09 × 100) / s* where *TA* = total acidity in grams of lactic acid per 100 mL of the sample, *v*= volume (mL) of NaOH used in titration, *N*= normality of the NaOH solution, *s*= volume (mL) of the sample used in the estimation, and 0.09 = lactic acid milliequivalent. (4) Density, measured through a 50 mL pycnometer. (5) Viscosity, estimated through an Ostwald viscosimeter by recording the time the sample took to reach different marks of the viscosimeter. We calculated the viscosity using the formula: *n_1_= (d_1_ × t_1_ × n_2_) / (d_2_ × t_2_)*, where *n_1_* and *n_2_* are the viscosities, *t_1_* and *t_2_* are the times taken by the flow, and *d_1_* and *d_2_* the densities of the liquid studied and water, respectively. (6) Percentage of alcohol: We distilled 200 mL of each sample using a distillation balloon flask and glass beads heated at 80 °C to complete evaporation, recovering 85–90% of the original volume. Then, using the recovered liquid we measured the percentage of alcohol with an alcoholometer.

### 2.4. Microbiological Characterization of the Beverages through Colony-Forming Units (CFU) 

In order to characterize the structure of the microbial community for each fermentation phase (FS, BS, P and IP), we used three media for cultivating CFU: (1) Tryptic Soy Agar, pH 7.3, a general medium that favours the development of a great variety of microorganisms; (2) Man, Rogosa and Sharpe (MRS) Agar, pH 6.5, a selective medium for lactic acid bacteria, with ammonium citrate that prevents the growth of Gram-negative bacteria; and (3) Sabouraud Dextrose (SD) Agar, pH 5.6, a selective medium for cultivation of fungi and yeasts, with antibiotics to inhibit growth of bacteria.

Each sample was diluted through a series of decimal dilutions until reaching l0^−6^, and 0.1 mL per sample was sown per 10 cm diameter petri dish, with three replicates per sample and culture medium.

Cultures were incubated for 72 h at 26 °C, and then characterization and morphotype counting were conducted. Each morphotype was characterized through attributes such as form, border, elevation, surface, colour, and light reflectance.

### 2.5. Statistical Analyses 

We conducted one-way and two-ways analyses of variance (ANOVA) and Tukey’s multiple range tests to evaluate differences among treatments, and principal component analysis to evaluate differentiation patterns associated with all variables studied. All data analyses were conducted with R software (v. 3.5.0).

## 3. Results

### 3.1. Sap Management for Pulque Production

**Figure 2:**
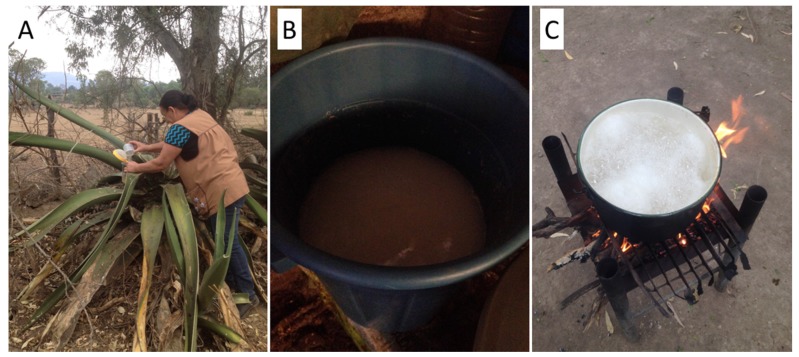
**(A**) Extraction of fresh sap *“aguamiel”* in SU in the species *A. salmiana* var. *salmiana*. (**B**) Inoculum for the pulque, “foot of pulque”, in a plastic barrel in T. (**C**) Boiled sap in SU.

Pulque producers at both sites collect sap from 8 to 10-year-old agaves, just when the meristem starts producing inflorescence. At that point, the producers cut the meristem and dig a cavity where the sap flows and accumulates. Every day, the producers collect the accumulated sap with a cup and a bucket and scrap the cavity to allow the sap to continue to flow. The producers have 10 to 15 agaves in production and collect on average 32 L of sap in T and 26 L in SU per day (Figure 2A). 

Differences in the way the sap is managed were recorded among sites. In T, after the sap is collected, it is transported to the homes of the producers and stored in spaces specially designed for the creation of pulque. The creation of pulque in T occurs in clean rooms, with little exposure to sunlight, and the people here consider it convenient to maintain low temperatures for a good preservation of the beverage. In these spaces, the producers have 20 L plastic barrels where they prepare the pulque. Additionally, the producers have a little bottle for maintaining the inoculum of the pulque, which is the remaining sediment from pulque prepared the day before. The producers use this to inoculate the fresh sap and, according to the people interviewed, for enhancing *“the aguamiel to work fast and making pulque tasty”*. 

To prepare the inoculum, the producers collect 3 L of aguamiel and leave it resting in a container covered with a blanket, causing the sap to undergo “spontaneous” fermentation. The liquid is allowed to ferment for 48 to 72 h. Later, this highly fermented aguamiel will be combined with fresh aguamiel in a ratio of 1:2 (fermented aguamiel and fresh aguamiel, respectively) for the first round of pulque production. After selling or consuming the pulque, a whitish sediment remains in the container, which may be perceived as slightly sandy; this is the inoculum, which people use to continue producing pulque on successive days (Figure 2B).

When adding the inoculum to the fresh sap, the people pour the sap through a mesh in order to remove the remains of the scraped tissue or insects that may fall into it. Once the fresh sap is added, the resulting mixture starts fermenting, producing an effervescence and a white foam. According to the producers, after 3 hours of fermentation, the pulque is ready for consumption or to be sold.

In SU, after the fresh sap is collected, it is strained and placed in a pot over a fire to slightly boil it for no more than 1 minute (Figure 2C). As soon as the aguamiel begins boiling, the people remove it from the heat and let it cool for 1 hour, then mix it with the inoculum. The effervescence of the beverage takes place after 2 hours, at which point the producers consider the pulque ready to be consumed and commercialized. The process of saving the inoculum in SU is like that practiced in T, but in this village, people leave the sap to ferment for a couple of days, and then mix this “foot of pulque” with the boiled sap. According to the pulque producers of SU, the sap generates an irritation called *“carame”*, and by boiling the sap they avoid carame.

### 3.2. Physical and Chemical Characteristics of Sap and Pulque

Table 2 shows the mean values and standard errors of the characteristics measured in the sap and pulque at different phases of fermentation at each study site. We identified significant differences in beverages among localities, and highly significant differences were identified based on the processes of sap boiling and inoculation (Table 2).

Concentrations of sugars (°Brix) in fresh sap were 9.8 ± 0.89 in SU and 9.57 ± 0.76 in T, whereas in pulque we recorded 8.22 ± 0.49 in SU and 7.48 ± 0.4 in T, and in the inoculum 5.47 ± 0.4 and 5.42 ± 0.29 in SU and T, respectively.

The beverage became progressively more acidic as fermentation advanced. Fresh sap had a pH of 6.23 ± 0.39 and 4.58 ± 0.13 in SU and T, respectively. The boiled sap of SU had a pH of 7.32 ± 0.58. Later, the pulque had a pH of 4.15± 0.14 and 3.94 ± 0.08, whereas the inoculum had a pH of 3.87 ± 0.13 and 3.69 ± 0.05, in SU and T, respectively. 

Lactic acid concentration (g/100 mL) in fresh sap was 0.23 ± 0.04 and 0.53 ± 0.06 in SU and T, respectively, increasing during fermentation to 0.82 ± 0.05 and 0.67 ± 0.06 in the pulque and 1.02 ± 0.03 and 0.77 ± 0.06 in the inoculum in SU and T, respectively. The boiled sap of SU had the lowest value of lactic acid (0.05 ± 0.02). 

Density (g/cm^3^) decreased with fermentation from 1.01 ± 0.001 and 1.01 ± 0.001 in fresh sap to 0.99 ± 0.007 and 0.98 ± 0.001 in pulque, and 0.97 ± 0.002 and 0.99 ± 0.003 in inoculum, from SU and T, respectively. Boiled sap had a density similar (1.02 ± 0.006) to non-boiled sap.

The viscosity (cP) of fresh sap was 1.22 ± 0.12 and 1.3 ± 0.09 in SU and T, respectively, and it increased with fermentation to 1.14 ± 0.03 and 1.59 ± 0.019 in pulque and 2.92 ± 0.16 and 2.48 ± 0.09 in the inoculum in SU and T, respectively. The viscosity of the boiled sap was 1.14 ± 0.03, similar to that of the non-boiled sap. 

The fresh sap in both localities had an alcohol content of 0%. When boiled, a small amount of alcohol was present, 0.53% ± 0.15, increasing to 3.88% ± 0.47 and 4.92% ± 0.29 in pulque in SU and T, respectively, and even more in the inoculum; 6.73% ± 0.75 in SU and 6.03% ± 0.35 in T.

### 3.3. Fermenting Microorganisms

**Figure 3:**
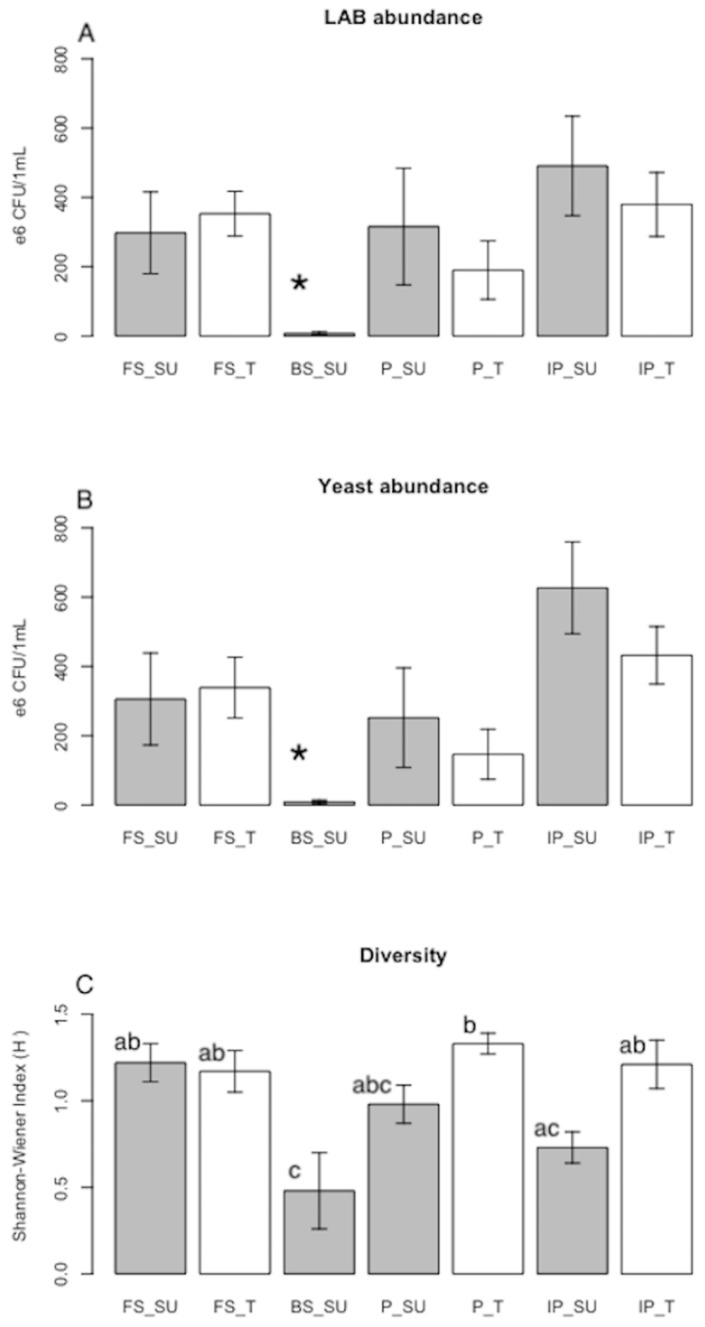
**(A**) Abundance of lactic acid bacteria (LAB). (**B**) Abundance of yeasts. (*) was a different treatment to the rest according to multiple Tukey tests (*p* < 0.05). (**C**) Diversity (Shannon–Wiener Index *H´*). Different letters in rows indicate significant differences according to Tukey tests (*p* < 0.05). Phases: fresh sap (FS), boiled sap (BS), pulque (P) and inoculum for pulque (IP). Sites: Santiago Undameo (SU), grey bars, and Tarímbaro (T), white bars.

Lactic acid bacteria (LAB) were recorded in the different phases of the beverage at both sites. In fresh sap of SU, we recorded 2.98 ±1.18 × 10^8^ colony-forming units per 1 mL (CFU), while 3.53 ± 0.65 × 10^8^ CFU were recorded in T. When the sap was boiled in SU, this decreased to 7.15 ± 4.55 × 10^6^ CFU, but in the pulque it increased markedly to 3.16 ± 1.68 ×10^8^ CFU in SU, while it increased to 1.9 ± 0.84 × 10^8^ CFU in T, where the fresh sap was not boiled. The highest abundance was recorded in the inoculum (4.91 ± 1.44 × 10^8^ CFU in SU and 3.80 ± 0.92 × 10^8^ CFU in T) (Table 3, Figure 3A). 

The pattern of yeast abundance was similar to that of LAB: 3.06 ± 1.33 × 10^8^ and 3.39 ± 0.88 × 10^8^ CFU in fresh sap from SU and T, respectively; a notable decrease was seen in boiled sap (8.72 ± 6 × 10^6^ CFU), then the levels recovered in pulque (2.52 ± 1.44 × 10^8^ CFU in SU and 1.47 ± 0.72 × 10^8^ CFU in T), and reached a maximum in the inoculum (6.27 ± 1.33 × 10^8^ CFU in SU and 4.32 ± 0.83 × 10^8^ CFU in T) (Table 3, Figure 3B).

In the general culture medium, we recorded the following abundances of yeast: 5.24 ± 1.17 × 10^8^ CFU in fresh sap of SU, and 8.49 ± 2.28 ×10^8^ in T, which decreased after boiling (16.56 ±10.04 × 10^6^ CFU). For pulque, we recorded 8.97 ± 4.79 ×10^8^ CFU in SU and 5.21 ± 1.51 ×10^8^ CFU in T, while for the inoculum we recorded 16.34 ± 6.11 ×10^8^ CFU for SU and 17.17 ± 3.92 ×10^8^ CFU for T. 

Although a clear pattern of CFU increase was seen during fermentation, with the greatest abundance of LAB and yeast found in the inoculum, the ANOVA showed no significant differences among most treatments or sites, except for boiled sap. 

In SU, LAB and yeasts are present in the fresh sap, but their abundance decreases in boiled sap. Then, when the boiled sap is inoculated, the inoculation promotes pulque production.

In beverages from T, where the fresh sap is inoculated with the inoculum to produce pulque, it is noted that the CFU of LAB and yeasts decreased in the pulque, which suggests that the colonies that were inoculated competed with those already occurring in the sweet sap. After fermentation reached the phase of pulque, the microorganisms continued growing, thus forming the inoculum that was used for the next inoculation (Figure 3).

The richness of LAB morphotypes recorded in fresh sap was 4.3 ± 0.7 (and 0.6 ± 0.2 after boiling) in SU and 4.3 ± 0.8 in T. In the pulque, we recorded 3 ± 0.5 in SU and 4.2 ± 0.9 morphotypes in T, as well as in the inoculum (2 ± 0.5 and 3.8 ± 0.5 morphotypes in SU and T, respectively). The richness of yeast morphotypes in fresh sap was 4.5 ± 0.6 (1 ± 0.4 after boiling) in SU and 4.5 ± 0.6 in T. For the pulque, we recorded 1.8 ± 0.3 in SU and 3 ± 0.6 morphotypes in T, whereas in the inoculum for the pulque we found 1.3 ± 0.3 and 2.3 ± 0.5 morphotypes in SU and T, respectively (Table 3). 

As fermentation progressed, a decrease in the morphotypes of both LAB and yeast was observed, with more occurring at the beginning of the process and less at the end. The lowest number of morphotypes was reported in boiled sap, because the abundance and richness of the microbial communities decrease with the effect of the temperature increase.

Regarding diversity, the reported values were *H´* = 1.22 ± 0.11 for fresh sap of SU, and *H´* = 1.17 ± 0.12 for T. For the pulque, it was *H´* = 0.98 ± 0.11 in SU, and *H´* = 1.33 ± 0.06 in T. Finally, in the inoculum, the diversity was *H´* = 0.73 ± 0.09 in SU and *H´* = 1.21 ± 0.14 in T. The lowest diversity was reported in the boiled sap, at *H´* = 0.48 ± 0.22 (Table 3, Figure 3C).

Pulque from T, from sap that had not been boiled, had a higher diversity than those from SU, apparently because the absence of boiling allows more morphotypes to be added from the inoculum to those already existing in fresh sap.

## 4. Discussion 

### 4.1. Pulque Management in the State of Michoacán: Similarities and Contrasts with Other Regions

The practices for preparing pulque reported here for Michoacán have particularities that differentiate them from other regions of Mexico. The cutting of agaves, the making of the cavity, and the sap extraction are very similar to methods practiced in other regions. In the region of central Mexico, in the states of Hidalgo and Tlaxcala, it is common that the extraction of the sap is not conducted with a cup, but with an *“acocote”*, the dried long fruit of *Lagenaria siceraria* which is used as a pipette to extract the sap [12,44]. 

The sap management systems of the areas in this study are similar in practice, but not in intensity, to those of other regions of Mexico. For instance, while in the studied localities of Michoacán, the producers handle from 10 to 15 plants and produce a maximum of 50 L of pulque per day; in Nanacamilpa, Tlaxcala, there are systems with extensive agave plantations, where a producer may have 40 hectares and nearly 1200 plants in production, generating up to 8000 L of pulque per day [12]. These more intense systems depend on the work of dozens of people who propagate, fertilize and cut the agaves, collect the sap, and create pulque with it [12], while in SU and T the production system is managed by household units [27].

Another notorious feature in the community of SU is the boiling of the sap, an uncommon practice in Mexico with no similar records available in other regions. However, an interesting case is that of the communities of the Ecuadorian Andes, where the practice of boiling the sap has been reported [45]. In that area, people make use of *A. americana,* using its flower buds and leaves as medicine, fiber, and fodder for cattle, as well as consuming the sap, called *“mishki”*, which means sweet in Kichwa. In addition to the consumption of the fresh sap of *A. americana*, a fermented beverage called *“guarango”* or *“chaguarmishki”* is prepared by boiling the sap and mixing it with the *“chaguarmishki”* of the previous days [45], similar to how pulque is prepared in SU.

The origin of *A. americana* and its history of use as a beverage in the equatorial region of the Andes is unclear, but the boiling of sap to prepare the fermented beverage might indicate it is a pre-Columbian activity, or was introduced by the Spanish conquerors [45]. The cultural parallelism behind boiling the sap between the *“chaguasmishki”* producers of the Ecuadorian Andes region and the pulque producers of the center of Michoacán should be further studied. A comparative analysis of the history of the management of agave sap between the two cultural groups may elucidate the reasons for boiling the sap before fermentation in these geographically distant regions—a practice that is not reported in any other region of Mexico.

The peculiarity of a short pulque fermentation of only a few hours in the two localities studied and the practice of boiling sap in SU could have important implications for the organoleptic profile of the beverage. Organoleptic characteristics are properties that are perceived with the senses of smell and taste; these are the criteria for the decision to consume or not consume a particular beverage.

In summary, a gradual change in the evaluated characteristics was reported during the phases of pulque preparation. Concentration of sugars (°Bx) showed a clear decreasing trend during fermentation. This is because of the consumption of sugars by the fermenting microorganisms—both those associated with the plant and those inoculated by people. Boiled sweet sap had a slightly higher concentration of sugar than non-boiled, but the difference was not significant. While the measured sugar concentrations were not significantly different, pulque from the boiled sap from SU was slightly sweeter than the unboiled sap from T and had a different sugar concentration with respect to the “foot of pulque” (phases that were not significantly different in T). An increase of the sugar concentration associated with boiling could be due to hydrolysis of compounds, like saponins; these are secondary metabolites present in agaves that contribute to defence against herbivores; with the increasing temperature saponins could discompose and release sugars [46,47]. 

The beverage became progressively more acidic as fermentation advanced, and pH and the concentration of lactic acid increased in the beverage, due to the metabolism of LAB; they consume the glucose of the fresh sap and generate pyruvate and consequently lactic acid [48]. Boiled sap had the most alkaline value of the samples, as well as the lowest concentration of lactic acid; this is because with the increase of the temperature during boiling, the lactic acid volatilizes, thus reducing the acidity of the beverage.

An increase of viscosity during fermentation indicates the accumulation of extracellular polysaccharides (EPS) suspended in the beverage, especially in the inoculum. Escalante et al. [26] reported an increased viscosity during the fermentation process, passing from 0.0012 K (Pas ^n^) in fresh sap to 0.0142 K (Pas ^n^) in pulque. 

A similar pattern was described for the alcohol content, which gradually increased with fermentation, with the inoculum having the highest values. This is the metabolic result of yeasts, especially those of the genus *Saccharomyces* [14].

The pulque production process in both locations takes 1 day; sap is collected in the morning, mixed with the inoculum, and after fermenting for a few hours it is consumed. The following day the process is repeated, the producer conducts a unique inoculation of the fresh sap, unlike in other regions of Mexico where the process to prepare pulque is prolonged for 3 to 5 days, as in Hidalgo or in Tlaxcala. In these regions, fresh sap is mixed with the inoculum, before letting it ferment for a few hours and later returning to add fresh sap to the mixture, to then let it ferment again. The following day the procedure is replicated. This is performed for several days, until the pulque is “mature”, meaning that it has desirable characteristics [12]. The process of several days of fermentation of the pulque generates a less sweet and slightly astringent beverage due to the start of acetic fermentation, in which bacteria like *Acetobacter* spp. and *Gluconobacter* spp. convert alcohol into acetic acid [34]. Additionally, the resulting pulque is considerably more viscous than that consumed in the localities studied in Michoacán, due to the accumulation of EPS during the longer preparation time.

### 4.2. Importance of the Fermenting Microorganisms

Communities of fermenting microorganisms are crucial for understanding the characteristics of the beverages. Escalante et al. [26] reported information about the abundance of CFU of LAB for fresh sap and pulque from Huitzilac, in the state of Morelos, while Valadez-Blanco et al. [43] reported this information for three communities of the state of Oaxaca (Table 3), which makes possible a comparison with the results of our study. Unfortunately, these studies do not report information about the *Agave* species studied, nor about the conditions of pulque preparation.

When comparing how LAB abundances change from the fresh sap phase to the pulque phase, the inter-phase abundances were found to be similar in San Jerónimo-Oaxaca and Santiago Undameo-Michoacán. In Los Arcos-Oaxaca, Huitzilac-Morelos and Tarímbaro-Michoacán, LAB abundances were reduced from the fresh sap phase to the pulque phase, most notably in the Los Arcos-Oaxaca area, and a very similar pattern of LAB decrease was observed when passing from fresh sap to pulque both in Huitzilac-Morelos and in Tarímbaro-Michoacán. In La Plazuela-Oaxaca on the other hand, there was a higher increase of LAB between the fresh sap to the pulque phase (Table 3).

The increased CFU of LAB in Santiago Undameo and decrease in Tarímbaro is apparently associated with fresh sap boiling. This pattern, and the high variation in other regions, suggest that documenting management practices of pulque are crucial for understanding what happens with microorganism communities. 

The presence of these microorganisms is important since, in addition to fermentation, they confer health benefits; LAB, for instance, are probiotic generating nutritious compounds, helping in the assimilation of other nutrients and may have anti-inflammatory properties and confer protection against pathogens by producing bacteriocins [15,49]. 

Cervantes–Elizarrarás et al. [40] evaluated the probiotic capacity of eight LAB strains from pulque (*Lactobacillus plantarum*) and two from fresh sap (*Pediococcus acidilactici*); their results showed a survival rate between 62%–96% in gastric juices and a rate of 53%–67% in bile salts and pancreatin. Moreover, 60% of the strains have antibacterial activity against *Escherichia coli* and *Staphylococcus aureus*, and all the strains have an inhibitory capacity against *Helicobacter pylori.*

Although the methodological approach of this study does not allow characterization of microorganisms at the species level, it provides important information about the behaviour of different functional groups, which makes it possible to isolate the most relevant morphotypes for further identification. 

Villareal–Morales et al. [38] recorded six yeast species (mainly *Kluyveromyces* spp.) and 11 bacteria strains (mainly *Lactococcus* spp.) from the fresh sap of *A. salmiana* in Coahuila, in the north of Mexico. Escalante et al. [26] identified 21 species of microorganisms in sap and pulque from Morelos (center of Mexico), with *Leuconostoc mesenteroides* and *Lactobacillus acidophilus* standing out in pulque fermentation. These records prove the high microbial diversity associated with each region. 

### 4.3. Mexican Norms for Pulque Flavor and Identity

The Mexican norms NOM-199-SCFI-2017 [50], NMX-V-037-1972 [51], and NMX-V-022-1972 [52] have established ranges of physical and chemical characteristics of fresh sap and pulque for commercialization (Table 4 and Table 5). In our opinion, these norms should be more specific to the *Agave* species (even varieties), the management practices involved, and the environmental and fermentation conditions. 

In Table 4 and Table 5, it is possible to see that the concentrations of sugar, acidity, lactic acid, and percentage of alcohol in the fresh sap and pulque reported by different studies may differ, even with the Mexican norms, indicating that variation is not well-represented in the norms. Other characteristics like density and viscosity should also be included as indicators of the quality of the beverages.

For example, the °Brix values reported fall within the range of the norm, except for the sap reported by Valadez–Blanco et al. [43]. For the pH, none of the cases fell within the range established by the norm, except the sap reported by Sánchez–Marroquín and Hope [53]. A similar consideration may be extended to lactic acid, for which all samples were below the levels established by the norm, including the slightly fermented sap (Table 4).

With pulque, a similar pattern occurs (Table 5). For °Brix, the norm establishes an interval between two and five, an interval with values lower than all reported, which implies that the norm accepts a pulque with a longer fermentation time, containing less sugar. Regarding the pH and lactic acid, it happens that the range of the norm is very limited, although in the different pulques reported, the variation in pH is minimal, the pulques could easily fall outside of the norm. The same occurs for lactic acid; the value reported by Sánchez–Marroquín and Hope [53] is slightly below the norm, and the reported value for SU is above. The percentages of alcohol also fluctuate among those registered, from pulques with low alcohol content (1.32% in Valadez–Blanco et al. [43], well below the norm), to others that fluctuate within the interval. The norm considers 7.5% alcohol as acceptable—a value that we reported for the phase inoculum, but not for pulque (Table 5).

Boiling fresh sap is not a common practice, but the pattern reported here suggests that it deserves more studies to analyze the causes for an increase of sugars, particularly the effect of boiling on saponins. Previous studies have documented that saponin content may be associated with plant management. For instance, Figueredo–Urbina et al. (2018) [54] reported lower amounts of saponins in cultivated plantations of *Agave inaequidens* and *A. cupreata* compared with wild populations of those species. Pulque produced from non-boiled sap has an increased sugar content, but boiling removes the irritation attribute of the beverage. We hypothesize that both properties result from the same action, but more studies are required in this direction.

In addition to the possible resulting attributes, boiling the sap is a factor of cultural identity of people from SU, as expressed by the producers: *“Here, we prepare well pulque; the sap is boiled, differently to other sites”*. Similarly, in other sites, the conditions of sweetness, viscosity, and acidity are particular, and moreover the addition of fruit (e.g., *Opuntia* spp., *Ananas comosus*, *Diospyros digyna*), leaves (*Ruta graveolens*, *Mentha spicata*), seeds (*Schinus mole*) or roots (*Acacia angustissima*) to prepare especially flavoured beverages makes the pulque ‘good’ by the standards of producers and consumers [12,20,55].

In addition to organoleptic attributes, the people of Santiago Undameo considered that boiling the sap allows prolonged storage time of the sweet sap and, therefore, extends its availability. In addition, this practice may help to improve conditions for hygienic preparation of the beverage, because it is possible avoid the presence of unwanted microorganisms, such that only the microbiota present in the “foot of pulque” are added. 

## 5. Conclusions

The practices of pulque preparation are similar among the studied communities, with the main difference being sap boiling in SU, which modifies the physical and chemical characteristics of the medium and affects the abundance of microorganisms. However, the differences become slighter in the other phases of pulque (P) and inoculum of pulque (IP). In the communities studied, the pulque preparation involves: 1) generating specific organoleptic profiles of flavour (acidity through fermentation and sweetness by sap boiling), odours and textures (lightly viscous pulque, as it only ferments for a few hours); 2) managing emerging attributes like alcohol concentration and the nutritional effects of LAB and yeasts present in the beverage; 3) increasing the period of preservation by boiling the sap and thereby removing the microorganisms, the sap can be stored without fermenting and increase its temporary availability; and 4) unique human cultural expressions in the form of preparing the beverage according to local values and preferences, selected by producers through management practices, finding contrasts between regions (e.g., Tlaxcala and Michoacán) and between communities in the same region (Santiago Undameo and Tarímbaro). In different regions of Mexico, general patterns of preparation of pulque can be identified, but the different preparation techniques, agave species and environmental contexts may determine constellation of fermentation conditions. The resulting diversity of qualities and types of products may involve in turn diversification and specialization of the microorganism consortia, but testing such a hypothesis requires a vast amount of research that would be of great importance for using and conserving these important genetic resources.

## Figures and Tables

**Table 1:** Examples of fermented beverages in Mexico, the species used in their creation, fermentation materials, and the regions where they are produced and consumed.

Fermented Beverage	Species	Fermented Substrate	Region of Mexico	References
**Tepache**	*Spondias* spp. or *Ananas comosus*	Fruit with peel	Center and South	[20]
**Colonche**	Columnar cacti or *Opuntia* spp.	Fruit without peel and seeds	Arid areas in the Center and North	[20,21]
**Pozol**	*Zea mays*	Nixtamalized and ground grains	Southeast and Yucatan Peninsula	[25]
**Tesgüino**	*Zea mays*	Germinated grains	North and Northwest	[23]
**Balche**	*Lonchocarpus longistylus*	Bark	Southeast and Yucatan Peninsula	[6,22]
**Taberna**	*Acrocomia aculeata*	Sap	Southeast	[19,20]
**Tuba**	*Cocos nucifera*	Sap	Southwest coast	[6,24]
**Pulque**	*Agave* spp.	Sap	Arid and temperate areas in the Center and North	[12,26,27]

**Table 2:** Physical-chemical characteristics (mean value ± standard error) of the fermentation phases of beverages in the localities Santiago Undameo (SU) and Tarímbaro (T).

Characteristic	Fresh Sap (FS)		Boiled Sap (BS)	Pulque (P)		Inoculum for Pulque (IP)	
	SU	T	SU	SU	T	SU	T
Concentration of sugar (°Bx) *	9.8 ± 0.89 (ab)	9.57 ± 0.76 (ab)	11.7 ± 0.3 (a)	8.22 ± 0.49 (b)	7.48 ± 0.4 (bc)	5.47 ± 0.4 (c)	5.42 ± 0.29 (c)
Acidity (pH) *	6.23 ± 0.39 (a)	4.58 ± 0.13 (b)	7.32 ± 0.58 (a)	4.15 ± 0.14 (b)	3.94 ± 0.08 (b)	3.87 ± 0.13 (b)	3.69 ± 0.05 (b)
Density (g/cm^3^) *	1.01 ± 0.001 (ab)	1 ± 0.003 (ab)	1.02 ± 0.006 (b)	0.99 ± 0.007 (ac)	0.98 ± 0.001 (cd)	0.97 ± 0.002 (d)	0.97 ± 0.003 (d)
Lactic acid (gr/100 mL) *	0.23 ± 0.04 (a)	0.53 ± 0.06 (b)	0.05 ± 0.02 (a)	0.82 ± 0.05 (cd)	0.67 ± 0.06 (bc)	1.02 ± 0.03 (d)	0.77 ± 0.06 (c)
Alcohol (%) *	0.00 ± 0 (a)	0.00 ± 0 (a)	0.53 ± 0.15 (a)	3.88 ± 0.47(b)	4.92 ± 0.29 (bc)	6.73 ± 0.75 (d)	6.03 ± 0.35 (cd)
Viscosity (cP) *	1.22 ± 0.12 (a)	1.3 ± 0.09 (a)	1.14 ± 0.03 (a)	1.59 ± 0.19 (a)	1.48 ± 0.13 (a)	2.92 ± 0.16 (b)	2.48 ± 0.09 (b)

^*^ significant differences *p* ≤ 0.001, different letters in rows indicate significant differences according to multiple Tukey tests (*p* < 0.05).

**Table 3:** Abundance of colony-forming units (CFU/mL), richness of morphotypes, and diversity (mean value ± standard error) of lactic acid bacteria (LAB) and yeast recorded for each fermentation phase and site.

	Fresh Sap (FS)						Boiled Sap (BS)	Pulque (P)						Inoculum for Pulque (IP)		
	SU	T	SJO	LAO	LPO	HM	SU	SU	T	SJO	LAO	LPO	HM	SU	T	HM
LAB abundance (CFU/mL) *	2.98 ± 1.18 × 10^8^ (a)	3.53 ± 0.65 × 10^8^ (a)	6.2 × 10^7^	3.7 × 10^9^	4.5 × 10^8^	3.2 × 10^8^	7.15 ± 4.55 × 10^6^ (b)	3.16 ± 1.68 × 10^8^ (a)	1.9 ± 0.84 × 10^8^ (a)	7.1 × 10^7^	8.3 × 10^7^	2.7 × 10^11^	1.5 × 10^8^	4.91 ± 1.44 × 10^8^ (a)	3.80 ± 0.92 × 10^8^ (a)	1.5 × 10^8^
LAB richness *	4.3 ± 0.7 (a)	4.3 ± 0.8 (a)	ND	ND	ND	ND	0.6 ± 0.2 (b)	3 ± 0.5 (ab)	4.2 ± 0.9 (a)	ND	ND	ND	ND	2 ± 0.5 (ab)	3.8 ± 0.5 (a)	ND
Yeast abundance (CFU/mL) *	3.06 ± 1.33 × 10^8^ (a)	3.39 ± 0.88 ×10^8^ (a)	ND	ND	ND	3.1 × 10^4^	8.72 ± 6 × 10^6^ (b)	2.52 ± 1.44 × 10^8^ (a)	1.47 ± 0.72 × 10^8^ (a)	ND	ND	ND	8.8 × 10^6^	6.27 ± 1.33 × 10^8^ (a)	4.32 ± 0.83 × 10^8^ (a)	1.4 × 10^7^
Yeast richness *	4.5 ± 0.6 (a)	4.5 ± 0.6 (a)	ND	ND	ND	ND	1 ± 0.4 (b)	1.8 ± 0.3 (b)	3 ± 0.6 (ab)	ND	In order toND	ND	ND	1.3 ± 0.3 (b)	2.3 ± 0.5 (ab)	ND
Diversity (*H’*) *	1.22 ± 0.11 (ab)	1.17 ± 0.12 (ab)	ND	ND	ND	ND	0.48 ± 0.22 (c)	0.98 ± 0.11 (abc)	1.33 ± 0.06 (b)	ND	ND	ND	ND	0.73 ± 0.09 (ac)	1.21 ± 0.14 (ab)	ND

SU: Santiago Undameo, Michoacan; T: Tarímbaro, Michoacan for this study; SJO: San Jerónimo, Oaxaca; LAO: Los Arcos, Oaxaca; LPO: La Plazuela, Oaxaca for Valadez–Blanco et al. [43]; HM: Huitzilac Morelos for Escalante et al. [26]. * Significant differences *p* ≤ 0.001. Different letters in rows indicate significant differences according to multiple range tests of Tukey (*p* < 0.05), only for samples of this study.

**Table 4:** Physical-chemical characteristics of fresh sap recorded in different studies, and the range of the Mexican norms (NMX).

Species	Locality	Concentration of Sugar (°Bx)	Acidity (pH)	Lactic Acid (g/100 mL)	Reference
*Agave* sp.	ND	11	7	0.18	[53]
*A. mapisaga*	Lomas de Romero, Puebla	ND	4.5	0.5	[35]
*Agave* sp.	Tamazulapan, Oaxaca	16	4.3	0.68	[43]
*A. atrovirens*	Michoacán	11.1	6.29	0.6	[36]
*A. atrovirens*	Las Mangas, Coahuila	9.55	6	ND	[34]
*A. salmiana*	Las Mangas, Coahuila	9.85	5.73	ND	[34]
*A. salmiana* var. *salmiana*	Santiago Undameo, Michoacán	9.8	6.23	0.23	This study
*A. mapisaga*	Tarímbaro, Michoacán	9.57	4.58	0.53	This study
Range NMX		8–12	6.6–7.5	0.9–1.03	[50,51,52,53]

ND = No Data.

**Table 5:** Physical-chemical characteristics of pulque recorded in different studies and the range of the Mexican norms (NMX).

Locality	Concentration of Sugar (°Bx)	Acidity (pH)	Lactic Acid (g/100mL)	%Alcohol	Reference
ND	6	4.6	0.348	5.43	[53]
Tamazulapan, Oaxaca	7.4	3.8	ND	1.32	[43]
Santiago Undameo, Michoacán	8.22	4.15	0.82	3.88	This study
Tarímbaro, Michoacán	7.48	3.94	0.67	4.92	This study
Range NMX	2–5	3.5–4	0.4–0.7	4–7.5	[50,51,52]

ND = No Data.
